# Meta-Analysis on the Prevalence and Significance of Incidental Findings in the Thyroid Gland Using Other PET Radiopharmaceuticals Beyond [^18^F]FDG

**DOI:** 10.3390/ph18050723

**Published:** 2025-05-15

**Authors:** Cesare Michele Iacovitti, Domenico Albano, Alessio Rizzo, Arnoldo Piccardo, Marco Cuzzocrea, Gaetano Paone, Pierpaolo Trimboli, Giorgio Treglia

**Affiliations:** 1 Division of Nuclear Medicine, Imaging Institute of Southern Switzerland, Ente Ospedaliero Cantonale, 6500 Bellinzona, Switzerland; cesaremichele.iacovitti@eoc.ch (C.M.I.); marco.cuzzocrea@eoc.ch (M.C.); gaetano.paone@eoc.ch (G.P.); 2 Department of Nuclear Medicine, ASST Spedali Civili di Brescia and University of Brescia, 25123 Brescia, Italy; domenico.albano@unibs.it; 3 Department of Nuclear Medicine, Candiolo Cancer Institute, FPO-IRCCS, 10060 Turin, Italy; alessio.rizzo@ircc.it; 4 Thyroid Center, Department of Nuclear Medicine, Ente Ospedaliero Ospedali Galliera, 16128 Genoa, Italy; arnoldo.piccardo@galliera.it; 5 Faculty of Biomedical Sciences, Università della Svizzera Italiana, 6900 Lugano, Switzerland; pierpaolo.trimboli@eoc.ch; 6 Thyroid Unit, Clinic for Endocrinology and Diabetology, Ente Ospedaliero Cantonale, 6900 Lugano, Switzerland; 7 Faculty of Biology and Medicine, University of Lausanne, 1015 Lausanne, Switzerland

**Keywords:** thyroid, incidentaloma, incidental, PET, positron emission tomography, nuclear medicine, somatostatin, choline, PSMA, FAPI

## Abstract

**Background**: Meta-analyses on the prevalence and significance of thyroid incidentalomas at PET (TIP) are available only about [^18^F]FDG. Focal TIP at [^18^F]FDG PET is not rare and may be malignant lesions in about one-third of cases. The aim of this study is to perform a meta-analysis on the prevalence and clinical significance of TIP using other PET radiotracers beyond [^18^F]FDG. **Methods**: A comprehensive literature search of studies about TIP was carried out using four different databases, screened until 31 December 2024. Only original articles about TIP using radiopharmaceuticals other than [^18^F]FDG were selected. A proportion meta-analysis on the prevalence and clinical significance of TIP was carried out on a patient-based analysis using a random-effects model. **Results**: 21 studies (29,409 patients) were included in the meta-analysis. PET was performed using radiolabeled somatostatin analogues (SSA) [*n* = 5], choline [*n* = 6], prostate-specific membrane antigen (PSMA) [*n* = 7], or fibroblast activation protein inhibitors (FAPI) [*n* = 3]. The uptake pattern of TIP was described as focal, diffuse, or mixed/heterogeneous. The pooled prevalence of TIP was 5.6% for SSA-PET, 6.1% for choline-PET, 4.2% for PSMA-PET, and 3.6% for FAPI-PET. The final diagnosis of TIP with a diffuse pattern was a benign condition or represented a physiological uptake. Conversely, TIP with focal or mixed/heterogeneous pattern may represent a benign condition in most cases, but even a malignant lesion in 6–10% of cases. **Conclusions**: As for [^18^F]FDG, TIP using other radiopharmaceuticals is not rare. Most of them are benign, but those with focal or heterogeneous uptake patterns may represent a malignant lesion in some cases (even if the risk of malignancy is lower compared to [^18^F]FDG PET), thus requiring further evaluation. Further studies are warranted to better clarify the clinical impact of TIP detection.

## 1. Introduction

Incidental imaging findings or incidentalomas are unexpected lesions diagnosed in patients undergoing imaging for an unrelated reason. The prevalence of incidentalomas in various organs is increasing due to the enhanced use and sensitivity of different diagnostic imaging modalities [1,2]. Notably, incidental imaging findings could be clinically relevant, but their prevalence and clinical significance vary depending on the anatomical site and the imaging method used [1,2].

Advancements in molecular imaging could lead to a more frequent identification of incidentalomas compared to morphological imaging, as functional abnormalities may precede morphological changes [1,2,3]. As for molecular imaging, positron emission tomography (PET), administering different radiopharmaceuticals, is increasingly used for oncological and non-oncological indications [4]. PET can be coupled with computed tomography (PET/CT) or magnetic resonance imaging (PET/MRI) as hybrid imaging methods, and different PET radiopharmaceuticals evaluating several metabolic patterns or receptor expression can be used [4].

About thyroid incidentalomas, they are found on imaging studies performed for reasons other than thyroid diseases and represent a common scenario encountered by health care providers [5,6,7]. The initial workup for thyroid incidentalomas comprises a thorough history and physical examination, thyroid function tests, thyroid ultrasound, and fine-needle aspiration of any suspicious lesions [5].

A systematic review is the application of strategies that limit bias in the assembly, critical appraisal, and synthesis of all relevant studies on a specific topic. Meta-analysis is the statistical synthesis of data from separate but comparable studies, leading to a quantitative summary of the pooled results [8,9]. Systematic reviews and meta-analyses can be applied to nuclear medicine, radiology, and hybrid imaging [10,11,12,13,14,15,16,17,18,19,20,21].

Several systematic reviews and meta-analyses have already evaluated the prevalence and clinical significance of thyroid incidentalomas detected by PET (TIP) using fluorine-18 fluorodeoxyglucose ([^18^F]FDG), a radiolabeled glucose analogue commonly used to detect tumours and inflammatory/infectious diseases characterized by increased glucose metabolism [22,23,24,25,26,27,28,29]. Available evidence-based data on TIP at [^18^F]FDG PET demonstrate that they are not rare, with a pooled prevalence ranging from 1 to 3% of all [^18^F]FDG PET scans [30]. On the other hand, TIP with diffuse [^18^F]FDG uptake patterns are usually benign conditions, representing thyroiditis in most of the cases [31]; TIP with focal [^18^F]FDG uptake patterns may have a risk of malignancy ranging from 20% to 35%, thus requiring further evaluation [30].

In current clinical practice, PET/CT and PET/MRI can be performed with other radiopharmaceuticals beyond [^18^F]FDG evaluating different metabolic pathways or receptor status, for instance radiolabeled somatostatin analogues to evaluate tumours with increased somatostatin receptor (SSTR) expression, radiolabeled choline to evaluate tumours or conditions with increased cell membrane turnover, radiolabeled prostate-specific membrane antigen (PSMA) ligands to evaluate mainly prostate cancer and fibroblast activation protein inhibitors (FAPI) to assess the activity of cancer-associated fibroblasts (CAFs) in several tumours [4].

**Figure 1:**
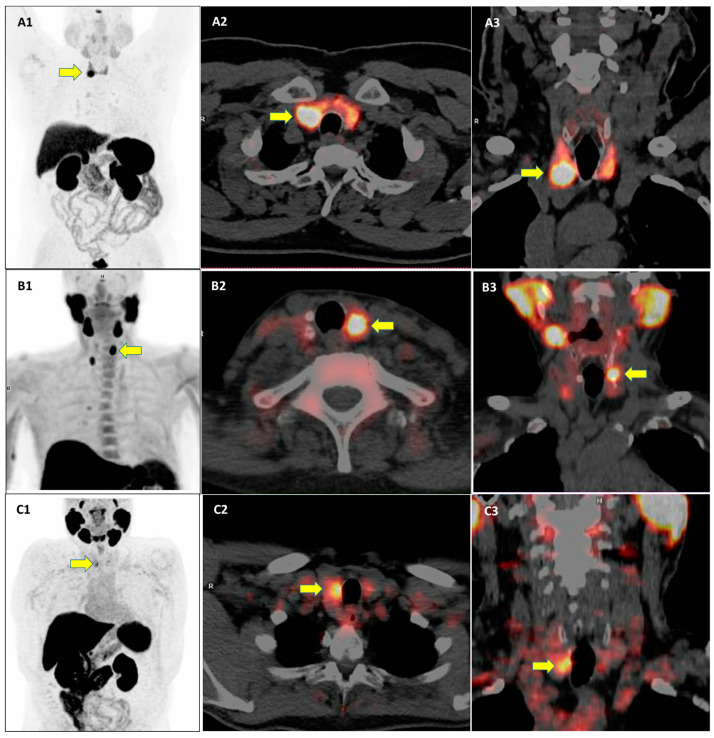
Examples of three cases of thyroid incidental findings (yellow arrows) at PET/CT using different PET radiopharmaceuticals. PET/CT images using [^68^Ga]Ga-DOTA-peptides (**A1**–**A3**), radiolabeled choline (**B1**–**B3**), and radiolabeled prostate-specific membrane antigen (**C1**–**C3**) are provided.

Interestingly, the prevalence and clinical significance of TIP with other PET radiopharmaceuticals beyond [^18^F]FDG have not been fully evaluated. Some examples of TIP using other radiopharmaceuticals beyond [^18^F]FDG are reported in Figure 1.

The aim of this systematic review and meta-analysis is therefore to evaluate the current literature to establish the prevalence and the clinical significance of TIP using other PET radiopharmaceuticals beyond [^18^F]FDG through a pooled analysis. Our hypothesis is that the prevalence and clinical significance of TIP could be quite different based on the PET radiopharmaceutical used.

## 2. Methods

### 2.1. Review Question, Working Group, and Review Protocol

The first step of this review was to formulate a review question defining patients, intervention, and outcomes. The review question was the following: “Which are the prevalence and clinical significance of thyroid incidentalomas at PET using other radiopharmaceuticals beyond [^18^F]FDG?”.

The working group for this study was composed of one junior nuclear medicine physician (C.M.I.), six senior nuclear medicine physicians (D.A., A.R., A.P., M.C., G.P., G.T.) with experience in PET and nuclear medicine in thyroid diseases, one senior radiologist (A.P.), and one senior endocrinologist with a special interest in thyroid disorders (P.T.). Five co-authors have extensive experience in systematic reviews and meta-analyses of diagnostic imaging techniques, including previous pooled analyses on prevalence and clinical significance of incidental imaging findings (D.A., A.R., A.P., P.T., and G.T.).

A predefined protocol was followed to perform this study [32], and the review article was reported according to the PRISMA statement [33]. The protocol was not published on PROSPERO or other public databases, as this is not mandatory [33].

### 2.2. Search Strategy

A comprehensive literature search of studies about TIP was carried out by two review authors independently (C.M.I. and G.T.). Four different databases (PubMed/MEDLINE, EMBASE, Cochrane Library, Google Scholar) were screened until 31 December 2024. Based on the review question, the following search string was created combining several text words: (A) “thyroid” AND (B) “incidental” OR “incidentaloma*” OR “incidental*” OR “unexpected” OR “unusual” AND C) “PET” OR “positron”.

No filters or restrictions on publication dates or language were used during the systematic literature search. To obtain a more sensitive literature search, the references of the potentially eligible articles were also screened for additional studies.

### 2.3. Study Selection

Two review authors independently performed the study selection (C.M.I. and G.T.), applying the predefined inclusion and exclusion criteria. Regarding inclusion criteria, studies or subsets of studies were included only if they investigated the prevalence and significance of TIP using other radiopharmaceuticals beyond [^18^F]FDG. The exclusion criteria were: (a) articles outside the scope of this review or not providing information on the prevalence and clinical significance of TIP; (b) review articles, editorials, comments, letters, and conference proceedings in the topic of interest; (c) case reports in the topic of interest.

Titles and abstracts of the records retrieved using the predefined search string in the selected databases were screened. After the exclusion of non-eligible records, the full texts of the potentially eligible articles were downloaded and screened. Finally, studies were included in the review after a virtual consensus meeting among four co-authors (C.M.I., D.A., A.R., and G.T.).

Articles included in the systematic review were included in the quantitative analysis (meta-analysis) on the specific radiopharmaceutical, only if sufficient data to calculate the prevalence and clinical significance of TIP were available.

### 2.4. Data Extraction and Quality Assessment

Two review authors (C.M.I. and G.T.) independently performed the data extraction and the quality assessment. The following data were extracted from the selected articles using predefined data collection forms: basic study characteristics, patient characteristics, technical aspects, and outcome data. The overall quality of the studies included in this systematic review was assessed online using the NIH quality assessment tools [34].

### 2.5. Statistical Analysis

The pooled prevalence of TIP using other radiopharmaceuticals beyond [^18^F]FDG was calculated for each radiopharmaceutical through a patient-based proportion meta-analysis using a random-effects model, which considers the variability among studies. Subgroup analyses taking into account the radiopharmaceutical uptake pattern were performed if sufficient data were available.

About the clinical significance of TIP, the pooled risk of malignancy was calculated, taking into account the final histopathological diagnosis.

Pooled data were presented with 95% confidence interval (95%CI) values.

Heterogeneity was estimated through the I^2^ index [18]. OpenMeta[Analyst] (version 1.0 for Windows) was used as free open-source statistical software.

## 3. Results

### 3.1. Literature Search

**Figure 2:**
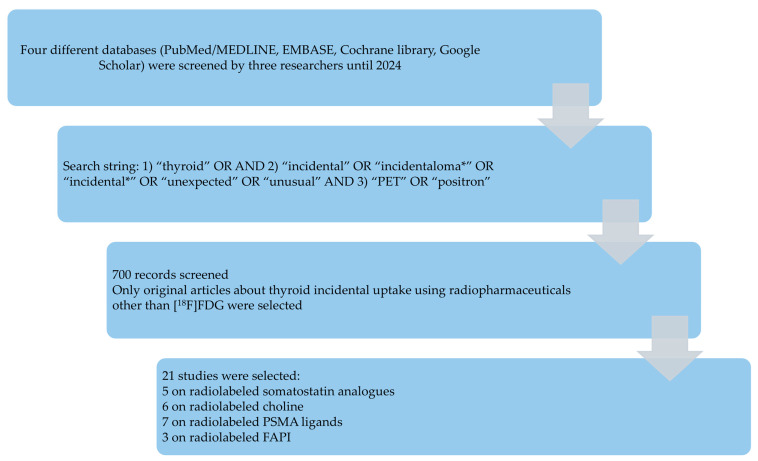
Results of the literature search.

Figure 2 summarizes the results of the literature search. A total of 700 records were identified using the selected databases. Titles and abstracts of these records were screened; 21 studies were finally included in the systematic review [35,36,37,38,39,40,41,42,43,44,45,46,47,48,49,50,51,52,53,54,55], and no additional studies were found screening the reference list of the retrieved articles. Five studies evaluated the prevalence and clinical significance of TIP with radiolabeled somatostatin analogues [35,36,37,38,39], six with radiolabeled choline [40,41,42,43,44,45], seven with radiolabeled PSMA ligands [46,47,48,49,50,51,52], and three with radiolabeled FAPI [53,54,55].

### 3.2. Qualitative Synthesis

Table 1 summarizes the main findings about original studies on TIP with other radiopharmaceuticals beyond [^18^F]FDG on 29,409 patients. The overall quality of the selected studies was assessed as moderate using the NIH quality assessment tool. Different radiopharmaceutical uptake patterns were described in TIP, including focal, diffuse, and heterogeneous/mixed patterns. Regarding the clinical significance of TIP based on the different radiopharmaceutical uptake pattern, when further evaluated, TIP with a diffuse uptake pattern usually represented a benign thyroid disease, whereas TIP with focal or heterogeneous uptake pattern could underlie a malignant thyroid disease in some cases [35,36,37,38,39,40,41,42,43,44,45,46,47,48,49,50,51,52,53,54,55].

#### 3.2.1. Radiolabeled Somatostatin Analogues

Five studies evaluated TIP at somatostatin receptor PET/CT or PET/MRI (using [^68^Ga]Ga-DOTA-peptides or [^64^Cu]Cu-DOTA-peptides), usually performed for the evaluation of neuroendocrine neoplasms [35,36,37,38,39]. The prevalence of TIP ranged from 2% to 11% of all PET scans. The prevalence of TIP with focal or heterogeneous uptake patterns ranged from 1% to 6% of all PET scans. When further evaluated, TIP with a diffuse uptake pattern at somatostatin receptor PET corresponded to thyroiditis, goitre, or absence of any clear thyroid disease due to physiological radiopharmaceutical uptake. Conversely, TIP with focal or heterogeneous uptake, even if most frequently benign, may represent malignant lesions in some cases. The overall risk of malignancy of TIP with focal or heterogenous uptake patterns ranged from 1% to 21%. The risk of malignancy of TIP with focal or heterogenous uptake pattern, further evaluated with thyroid imaging, ranged from 4% to 31%. At histopathological diagnosis, the malignant lesions described among TIP at somatostatin receptor PET corresponded to papillary thyroid carcinoma, medullary thyroid carcinoma, and metastases to the thyroid gland [35,36,37,38,39]. There was no significant difference in radiopharmaceutical uptake among benign and malignant TIP at somatostatin receptor PET [35,36,37,38,39].

#### 3.2.2. Radiolabeled Choline

Six studies evaluated TIP at radiolabeled choline PET/CT or PET/MRI (using [^11^C]Choline or [^18^F]Fluorocholine), usually performed for the evaluation of prostate cancer or for localization of hyperfunctioning parathyroid glands in patients with hyperparathyroidism [40,41,42,43,44,45]. The prevalence of TIP ranged from 1% to 13% of all PET scans. The prevalence of TIP with focal or heterogeneous uptake patterns ranged from 0.3% to 9% of all PET scans. When further evaluated, TIP with a diffuse uptake pattern at radiolabeled choline PET corresponded to thyroiditis, goitre, or absence of any clear thyroid disease due to physiological radiopharmaceutical uptake. Conversely, TIP with focal or heterogeneous uptake, even if most frequently benign, may represent malignant lesions in some cases. The overall risk of malignancy of TIP with focal or heterogenous uptake patterns ranged from 0 to 22%. The risk of malignancy of TIP with focal or heterogenous uptake pattern, further evaluated with thyroid imaging, ranged from 0 to 25%. At histopathological diagnosis, the malignant lesions described among TIP at radiolabeled choline PET corresponded to papillary thyroid carcinoma in most of the cases and less frequently to lymphoma, squamous cell carcinoma, and metastases to the thyroid gland [40,41,42,43,44,45]. There was no significant difference in radiopharmaceutical uptake among benign and malignant TIP at radiolabeled choline PET [40,41,42,43,44,45].

#### 3.2.3. Radiolabeled PSMA Ligands

Seven studies evaluated TIP at PET/CT or PET/MRI with radiolabeled PSMA ligands (using [^68^Ga]Ga-PSMA-11, [^18^F]F-DCFPyL, [^18^F]F-PSMA-JK-7, or [^18^F]F-PSMA-1007), usually performed for the evaluation of prostate cancer [46,47,48,49,50,51,52]. The prevalence of TIP ranged from 0.5% to 22% of all PET scans. The prevalence of TIP with focal or heterogeneous uptake patterns ranged from 0.5% to 6% of all PET scans. When further evaluated, TIP with diffuse uptake pattern at radiolabeled PSMA ligands PET corresponded to thyroiditis, goitre, or absence of any clear thyroid disease due to physiological radiopharmaceutical uptake. Conversely, TIP with focal or heterogeneous uptake, even if most frequently benign, may represent malignant lesions in some cases. The overall risk of malignancy of TIP with focal or heterogenous uptake patterns ranged from 0 to 25%. The risk of malignancy of TIP with focal or heterogenous uptake pattern, further evaluated with thyroid imaging, also ranged from 0 to 25%. At histopathological diagnosis, the malignant lesions described among TIP at radiolabeled PSMA ligands PET corresponded to papillary thyroid carcinoma in most of the cases and less frequently to follicular thyroid carcinoma, Hürthle cell carcinoma, and metastases to the thyroid gland [46,47,48,49,50,51,52]. A study showed that the prevalence of TIP may be different, taking into account the different radiolabeled PSMA ligands: a lower TIP prevalence was demonstrated using [^18^F]F-DCFPyL and [^18^F]F-PSMA-JK-7, and a higher TIP prevalence was found using [^18^F]F-PSMA-1007 [47]. Prevalence of TIP also varies, taking into account the different PET image analysis methods, being higher using visual analysis compared to semi-quantitative analysis [47]. Conversely, the prevalence of TIP did not differ significantly between observers [47]. The radiopharmaceutical uptake was significantly higher in patients with malignant findings than in patients with benign conditions [46,50].

#### 3.2.4. Radiolabeled FAPI

Only three studies evaluated TIP at PET/CT or PET/MRI with radiolabeled FAPI (using [^68^Ga]Ga-FAPI-46, [^68^Ga]Ga-FAPI-04 or [^18^F]F-FAPI-04), performed for the evaluation of several tumours [53,54,55]. The prevalence of TIP ranged from 1% to 20% of all PET scans. Only TIPs with diffuse radiopharmaceutical uptake were described. When further evaluated, TIP with diffuse uptake pattern at radiolabeled FAPI PET corresponded to thyroiditis, goitre, or absence of any clear thyroid disease due to physiological radiopharmaceutical uptake in most of the cases. A single malignant lesion was described among patients with diffuse uptake of radiolabeled FAPI in the thyroid gland corresponding to a lymphoma at histopathology [53,54,55].

### 3.3. Quantitative Synthesis

Meta-analyses on the prevalence of TIP demonstrated the following pooled values: 5.6% (95%CI: 3.0–8.2%) for PET with radiolabeled somatostatin analogues, 6.1% (95%CI: 2.6–9.5%) for PET with radiolabeled choline, 4.2% (95%CI: 2.7–5.7%) for PET with radiolabeled PSMA ligands, and 3.6% (95%CI: 0–7.5%) for PET with radiolabeled FAPI.

A subgroup analysis on the prevalence of TIP for focal and heterogeneous radiopharmaceutical uptake patterns demonstrated the following pooled values: 3.5% (95%CI: 1.9–5.1%) for PET with radiolabeled somatostatin analogues, 1.7% (95%CI: 0.7–2.7%) for PET with radiolabeled choline, 1.9% (95%CI: 1.1–2.6%) for PET with radiolabeled PSMA ligands, and not calculable for PET with radiolabeled FAPI.

Meta-analyses on the overall malignancy risk of TIP with focal or heterogeneous uptake pattern demonstrated the following pooled values: 5.7% (95%CI: 0.5–10.9%) for PET with radiolabeled somatostatin analogues, 10.1% (95%CI: 5.1–15%) for PET with radiolabeled choline, 6.5% (95%CI: 2.5–10.5%) for PET with radiolabeled PSMA ligands, and not calculable for PET with radiolabeled FAPI. These values slightly increased, considering only TIP further evaluated as reported in Table 1.

Significant statistical heterogeneity was found in the meta-analyses on the prevalence and clinical significance of TIP with different PET radiopharmaceuticals (I^2^ > 50%).

## 4. Discussion

### 4.1. Literature Data

To the best of our knowledge, this is the first comprehensive review and meta-analysis providing a summary on the prevalence and clinical significance of TIP using other PET radiopharmaceuticals beyond [^18^F]FDG. We have included only original studies in our review, excluding case reports, which provide low-quality evidence due to several biases [56].

Our review found that most literature on TIP focuses on radiolabeled somatostatin analogues, choline, and PSMA, with fewer studies available on TIP using other PET radiopharmaceuticals.

The thyroid gland is a frequent site of incidental radiopharmaceutical uptake with PET radiopharmaceuticals [3]. In our analysis, we have found that the overall pooled prevalence of TIP with other radiopharmaceuticals beyond [^18^F]FDG varies between 4% and 6% (quite similar to that of TIP using [^18^F]FDG); therefore, these findings are not rare and should not be overlooked.

Similarly to [^18^F]FDG PET, whereas diffuse tracer uptake pattern in the thyroid gland represents a benign condition in nearly all patients with TIP using other PET radiopharmaceuticals, focal or heterogeneous tracer uptake pattern in the thyroid gland may represent a malignant lesion, even if in a minority of patients. The overall pooled risk of malignancy of TIP with focal or heterogeneous uptake patterns ranged from 6% to 10% for most of the PET radiopharmaceuticals evaluated in our analysis. Notably, this risk seems to be significantly lower compared to the risk of malignancy of TIP with focal uptake pattern using [^18^F]FDG (reported to be between 20% and 35% according to evidence-based data [30]). Potential reasons for this difference among [^18^F]FDG and other PET radiopharmaceuticals could be related to the different uptake mechanisms of these PET radiopharmaceuticals. [^18^F]FDG uptake, reflecting the glucose metabolism, is more associated with the biological aggressiveness of the lesions compared to other PET radiopharmaceuticals. This could explain the higher malignancy risk of TIP using [^18^F]FDG compared to other PET radiopharmaceuticals. However, it is not possible to discriminate between benign and malignant TIP only at visual or semi-quantitative analyses of PET images with different radiopharmaceuticals due to the overlap of radiopharmaceutical uptake among these two groups [30,35,36,37,38,39,40,41,42,43,44,45,46,47,48,49,50,51,52,53,54,55].

Notably, due to the possible risk of malignancy of TIP with different PET radiopharmaceuticals, in particular in cases of focal or heterogeneous radiopharmaceutical uptake patterns, clinical evaluation, thyroid function test, and thyroid ultrasound should be suggested in the PET report to further evaluate TIP when they are detected [35,36,37,38,39,40,41,42,43,44,45,46,47,48,49,50,51,52,53,54,55]. Interestingly, current evidence suggests that investigation and management of thyroid nodules should not be influenced by the mode of detection (incidental versus non-incidental thyroid nodule) [57].

It could be hypothesized that if malignant thyroid lesions are incidentally detected using other PET radiopharmaceuticals beyond [^18^F]FDG, these PET radiopharmaceuticals could be used to evaluate primary thyroid malignancies. However, even if literature data are available on the use of radiolabeled somatostatin analogues [58,59,60,61,62,63], choline [64,65,66,67,68], PSMA ligands [62,69,70,71,72,73,74] and FAPI [74,75,76,77,78,79] in thyroid cancer; currently, these PET methods are used only in research setting for this purpose [30], except the use of somatostatin receptor PET in medullary thyroid cancer according to existing guidelines [80].

### 4.2. Limitations and Suggestions for Future Research

Several limitations of our analysis should be acknowledged. First, there is still a limited number of studies assessing TIP with each PET radiopharmaceutical beyond [^18^F]FDG. Second, significant heterogeneity among the studies included in the meta-analyses was found, likely due to differences in patient characteristics, countries (with different iodine status influencing the prevalence of thyroid diseases), PET radiopharmaceuticals, imaging protocols, and indications among the included studies. In this regard, some PET radiopharmaceuticals evaluated are used mainly for prostate cancer, and this could create a possible gender bias. Third, even if we have excluded case reports from the analysis, we cannot exclude the presence of publication bias; funnel plots were not used to assess the presence of publication bias due to the low number of studies available for each PET radiopharmaceutical. Both heterogeneity and publication bias could have an impact on the results presented in this study.

As a first suggestion for future research, we recommend performing more studies on the prevalence and clinical significance of TIP with other tracers beyond [^18^F]FDG and, in particular, with FAPI ligands. Second, it could be interesting to evaluate the possible change in management related to TIP detection with PET imaging using different PET radiopharmaceuticals. Third, long-term clinical outcomes of patients with TIP could be assessed in future studies.

## 5. Conclusions

As for [^18^F]FDG PET, TIP using other PET radiopharmaceuticals is not rare and should not be overlooked. Most of these findings are benign, but those with focal or heterogeneous radiopharmaceutical uptake patterns may represent malignant lesions in some cases (even if the malignancy risk is lower compared to [^18^F]FDG PET), thus requiring further evaluation. The evidence-based data from this analysis should inform future guidelines on managing thyroid incidentalomas. However, further studies are needed to better understand the clinical impact of TIP detection using various PET radiopharmaceuticals.

## Figures and Tables

**Table 1:** Main findings of studies on incidental thyroid findings at PET (TIP) with different radiopharmaceuticals.

PET Tracer	Ref.	Patients with TIP	Patients with Focal or Heterogeneous TIP	Patients with PET Scans	Prevalence of TIP	Prevalence of Focal or Heterogeneous TIP	Patients with Focal or Heterogeneous TIP Further Evaluated	Patients with Verified Malignant Focal or Heterogeneous TIP	Percentage of Malignant Focal or Heterogeneous TIP Among Those Further Evaluated	Overall Risk of Malignancy of Focal or Heterogeneous TIP	Final Diagnosis of Malignant TIP
Radiolabeled somatostatin analogues	[35]	42	42	1808	2.3%	2.3%	16	5	31.3%	11.9%	2 MTC + 3 metastasis
	[36]	61	44	837	7.3%	5.3%	33	2	6.1%	4.5%	2 PTC
	[37]	NA	80	1927	NC	4.2%	26	1	3.8%	1.3%	1 MTC
	[38]	26	14	237	11.0%	5.9%	10	3	30.0%	21.4%	3 PTC
	[39]	46	12	1150	4.0%	1.0%	12	1	8.3%	8.3%	1 PTC
Pooled values (95%CI)		175	192	5959	5.6% (3.0–8.2)	3.5% (1.9–5.1)	97	12	9.9% (1.8–18.0)	5.7% (0.5–10.9)	
Radiolabeled choline	[40]	25	17	195	12.8%	8.7%	17	2	11.8%	11.8%	2 PTC
	[41]	NA	103	10,047	NC	1.0%	74	9	12.2%	8.7%	7 PTC + 1 SCC + 1 lymphoma
	[42]	50	10	388	12.9%	2.6%	8	2	25.0%	20.0%	1 PTC + 1 metastasis
	[43]	3	1	368	0.8%	0.3%	1	0	0.0%	0.0%	-
	[44]	NA	9	368	NC	2.4%	8	2	25.0%	22.2%	2 PTC
	[45]	15	NA	1000	1.5%	NC	NA	0	NC	NC	-
Pooled values (95%CI)		93	140	12,366	6.1% (2.6–9.5)	1.7% (0.7–2.7)	108	15	13.4% (7.0–19.7)	10.1% (5.1–15.0)	
Radiolabeled PSMA ligands	[46]	67	36	769	8.7%	4.7%	36	4	11.1%	11.1%	4 PTC
	[47]	110	31	502	21.9%	6.2%	4	1	25.0%	3.2%	1 PTC
	[48]	61	43	5434	1.1%	0.8%	25	3	12.0%	7.0%	1 FTC + 1 HCC + 1 metastasis
	[49]	7	7	1445	0.5%	0.5%	6	0	0.0%	0.0%	-
	[50]	13	8	341	3.8%	2.3%	8	2	25.0%	25.0%	1 PTC + 1 metastasis
	[51]	9	6	438	2.1%	1.4%	6	0	0.0%	0.0%	-
	[52]	6	6	764	0.8%	0.8%	6	1	16.7%	16.7%	1 PTC
Pooled values (95%CI)		273	137	9693	4.2% (2.7–5.7)	1.9% (1.1–2.6)	91	11	11.7% (5.2–18.1)	6.5% (2.5–10.5)	
Radiolabeled FAPI	[53]	6	NA	556	1.1%	NC	NA	0	NC	NC	-
	[54]	4	NA	20	20.0%	NC	NA	0	NC	NC	-
	[55]	39	NA	815	4.8%	NC	NA	0	NC	NC	1 lymphoma
Pooled values (95%CI)		49	NA	1391	3.6% (0–7.5)	NC	NA	0	NC	NC	

Legend: FAPI = fibroblast activation protein inhibitors; FTC = follicular thyroid cancer; HCC = Hürthle cell carcinoma; MTC = medullary thyroid cancer; NA = not available; NC = not calculable; PSMA = prostate-specific membrane antigen; PTC = papillary thyroid cancer; SCC = squamous cell carcinoma; TIP = thyroid incidentalomas at PET.

## Data Availability

The data presented in this study are available on request from the corresponding author.
